# Mycelial compatibility, anastomosis, and nucleus numbers of eight Mexican *Hirsutella citriformis* strains isolated from *Diaphorina citri*

**DOI:** 10.7717/peerj.11080

**Published:** 2021-04-19

**Authors:** Orquídea Pérez-González, Ricardo Gomez-Flores, Patricia Tamez-Guerra

**Affiliations:** 1 Facultad de Ciencias Biológicas, Universidad Autónoma de Nuevo León, San Nicolás de los Garza, Nuevo Leon, Mexico

**Keywords:** Nit mutants, Nuclei, Fungus mexican native strains, Mycelial characterization

## Abstract

**Background:**

Among entomopathogenic fungi, *H. citriformis* has been recognized as potential biocontrol agent against the Asian citrus psyllid *Diaphorina citri* (Hemiptera: Liviidae). Nevertheless, this fungus is poorly characterized. Previous molecular studies have shown high sequence similarities among strains, but significant differences in *Diaphorina citri* virulence.

**Objective:**

The aim of the present study was to determine mycelial compatibility and anastomosis, and nucleus numbers in mycelium and conidia of eight *H. citriformis* strains isolated from mycosed *D. citri* adults collected from several Mexican states.

**Methods:**

Mycelial compatibility and anastomosis evaluation was performed after pairing strains, leading to 36 confrontations, and cultured in chlorate minimum medium to obtain mutants for vegetative compatibility group.

**Results:**

Hypha or conidia nuclei were visualized with safranin-O and 3% KOH, and 0.05% trypan blue–lactophenol solution. *H. citriformis* strains showed compatibly and anastomosis events after confrontation. In addition, they showed one nucleus per conidium and mycelium section. It was not possible to obtain *H. citriformis nit* mutants from the chlorate concentrations tested.

**Conclusions:**

To date, this is the first report demonstrating mycelial compatibility, anastomosis occurrence, and hyphae and conidia nuclei number among *H. citriformis* strains.

## Introduction

*Hirsutella citriformis* Speare is a poorly studied fungus, which has currently attracted interest because of its biocontrol potential against *Diaphorina citri* Kuwayama and *Bactericera cockerelli* Sulk. *D. citri* is a vector of the bacterium *Candidatus* Liberibacter spp., causing huanglongbing (HLB) in citrus (Halbert & Manjunath, 2004), whereas *B. cockerelli* is a vector of the *Candidatus* Liberibacter *solanacearum* (Liefting et al., 2008) phytopathogen bacterium associated with ‘zebra chip’ disease in potato. *H. citriformis* importance relies on that despite the fact that other entomopathogenic fungi have been reported infecting *D. citri,* such as *Beauveria bassiana* (Bals.) Vuill., *Isaria* (*Paecilomyces*) *fumosorosea* (Wize) Brown & Smith, and *Lecanicillium lecanii* Zare & Gams (Grafton-Cardwell, Stelinski & Stansly, 2013); this is the only *Hirsutella* species reported infecting *D. citri*, which has been shown to cause significant natural epizootics on this pest in field (Aubert, 1987; Subandiyah et al., 2000; Cabrera et al., 2004; Hall et al., 2012). Because of the characteristic sporulation on the insect (Hall et al., 2012), visual detection of infected *D. citri* specimens in the trees frequently suggests biological control potential.

*H. citriformis*, as well as other entomopathogenic fungi, does not have a known sexual state. This group represents the imperfect state of Cordyceps, Ophiocordyceps or Torrubiella (Tanada & Kaya, 1993). Genetic recombination in asexual fungi (parasexual cycle) is a potential source for genotypic diversity among fungi populations, including *H. citriformis*, which occurs after compatible vegetative hyphae are fused (Paccola-Meirelles & Azevedo, 1991; Couteaudier & Viaud, 1997; Bello & Paccola-Meirelles, 1998; Dalzoto et al., 2003). No changes were observed when fungus mycelium is crosslinked during growth, thus vegetative compatibility was not tested since all strains showed similar growth in different nitrogen sources. Crosslinking during growth is a random event that might result in virulence alteration against common pathogens or change the common variety of the host strain (Burdon & Silk, 1997).

*H. citriformis* reports have revealed significant variability, mainly in morphology, growth, conidiation, and pathogenicity (Mains, 1951; Subandiyah et al., 2000; Meyer, Hoy & Boucias, 2007; Casique-Valdes et al., 2011; Pérez-González et al., 2015a). Mexican strains used in the present study were isolated from *D. citri* mycosed adults showing synnemata and the insect specimens were collected from several states of Mexico (Pérez-González et al., 2015a). Isolates presented diverse macroscopic morphology, including size of reproductive structures, growth, color, and dimophism (Pérez-González et al., 2015a; Pérez-González et al., 2015b). In contrast, 28S gene phylogenetic analysis-based molecular characterization showed identical sequence among strains, whereas internal transcribed spacer (ITS) analysis demonstrated that Mexican strains were very similar to each other, with sequences variation lower than 0.21% (Pérez-González et al., 2015a). Nevertheless, after testing those strains virulence against *D. citri* adults under laboratory conditions, their insecticidal activity was variable, resulting in mortality ranges from 54.8% to 91.9% (Pérez-González et al., 2015a).

The aim of the present study was to determine mycelial compatibility and anastomosis, and nucleus numbers in mycelium and conidia of eight *H. citriformis* strains isolated from mycosed *D. citri* adults, collected from several Mexican states.

## Methods

### Fungi source

*H. citriformis* strains tested were provided by the National Institute of Forestry, Agriculture and Livestock (INIFAP) Experimental Station of General Teran, Nuevo Leon, and by the Biotechnology Institute of Biological Sciences School at Autonomous University of Nuevo Leon (IB-UANL), Mexico. INIFAP strain codes were INIFAP-Hir-1, INIFAP-Hir-2, INIFAP-Hir-4, INIFAP-Hir-5, INIFAP-Hir-6, and INIFAP-Hir-7. IB-UANL strain codes were IB-Hir-1 and IB-Hir-2.

### Culture medium

Strains were cultured on potato dextrose agar (PDA), water agar (WA), and water agar supplemented with 2% dextrose (WAD). Agar basal medium (MM) contained (per L) 30 g sucrose, 1 g KH_2_PO_4_, 0.5 g MgSO_4_.7H_2_O, 0.5 g KCl, 10 mg FeSO_4_.7H_2_O, 20 g agar, and 0.2 mL of a trace element solution containing (per 95 mL) 5 g citric acid, 5 g ZnSO_4_.7H_2_O, 1g Fe(NH_4_)_2_(SO_4_)_2_.6H_2_O, 0.25 g CuSO_4_.5H_2_O, 50 mg MnSO_4_.H_2_O, 50 mg H_3_PO_4_, 50 mg NaMoO_4_.2H_2_O, and 1.5%, 2.0%, 2.5% or 3.0% KClO_3_ (Becton Dickinson of Mexico, Mexico City, Mexico).

### Mycelial compatibility

Strains cultured on PDA, were confronted among them, evaluating all possible combinations (36 confrontations) in order to determine strains mycelial compatibility. Two mycelial discs collected from 14-day-old colonies, were placed in 100 × 15 mm Petri dishes with 25 mm to 35 mm separation distance between them. Plated were then incubated at 25 °C ± 2 °C for 3 wk to verify mycelium fusion. Confronted strains with potential to fuse their mycelia were considered compatible, whereas formation of a barrier between them was considered as incompatible strains. Experiments were performed three times with three replicate determinations per treatment.

### Anastomosis

For anastomosis experiments, strains were combined and placed on 76 × 26 mm sterile slides (Chance Propper Ltd., Smethwick, Warely, England) coated with a thin layer of WA, supplemented with 2% dextrose in a Petri dish at a distance of 2 ± 0.5 cm from each other. After 21–28 d incubation at 25 ± 1 °C, slide was taken from the plate and the original mycelium fragments were carefully removed, leaving only newly grown hyphae, which were stained with trypan blue-lactophenol [0.05 g of trypan blue solution in 100 mL of lactophenol (20 mL of 85% lactic acid and 20 g of phenol, 40 mL of glycerol, and 20 mL of distilled water)] (Burpee et al., 1978). A cover slip was placed on top of the developed hyphae and examined by light microscopy (Olympus CX41, Olympus, Mexico City). Anastomosis between strains was confirmed by visually tracing the anastomosing cells origin. Experiments were performed three times with three replicate determinations per treatment.

### Vegetative compatibility

Vegetative compatibility between strains was determined by testing non-utilizing nitrate mutants (Brooker, Leslie & Dickman, 1991). To select mutants, strain mycelial discs (0.5 mm diameter) of the monoconidial culture were used to inoculate on chlorate plates. Chlorate medium was prepared by adding 1.5%, 2.0%, 2.5%, and 3% potassium chlorate to the MM agar (MM-C). After 3 wk to 4 wk, thin fast-growing sectors were observed.

To phenotypically characterize *nit* mutants, MM was prepared using the following nitrogen sources: MM supplemented with 1.6 g/L ammonium tartrate (MM-TA), 2 g/L of nitrate (MM-N), 0.5 g/L nitrite (MM-NI), 0.2 g/L hypoxanthine (MM-H), or 0.2 g uric acid (MM-UA) (Correll, Klittich & Leslie, 1987). Colonies growth in chlorate plates were transferred to 60 mm ×15 mm Petri dishes containing MM supplemented with each of the nitrogen sources to detect *nit* mutant type, and incubated at 25 °C for 2 wk to 3 wk.

### Conidia and mycelium nuclear characterization

Hypha nuclei were stained with 3% KOH-safranin O (Bandoni, 1979) and 0.05% trypan blue–lactophenol solution. The culture technique was similar to that used by the anastomosis test. Conidia nucleus preparations were visualized with the same stain used for mycelium nuclei. Slides were then examined by light microscopy (Olympus, Mexico City).

## Results

### Mycelial compatibility

**Figure 1:**
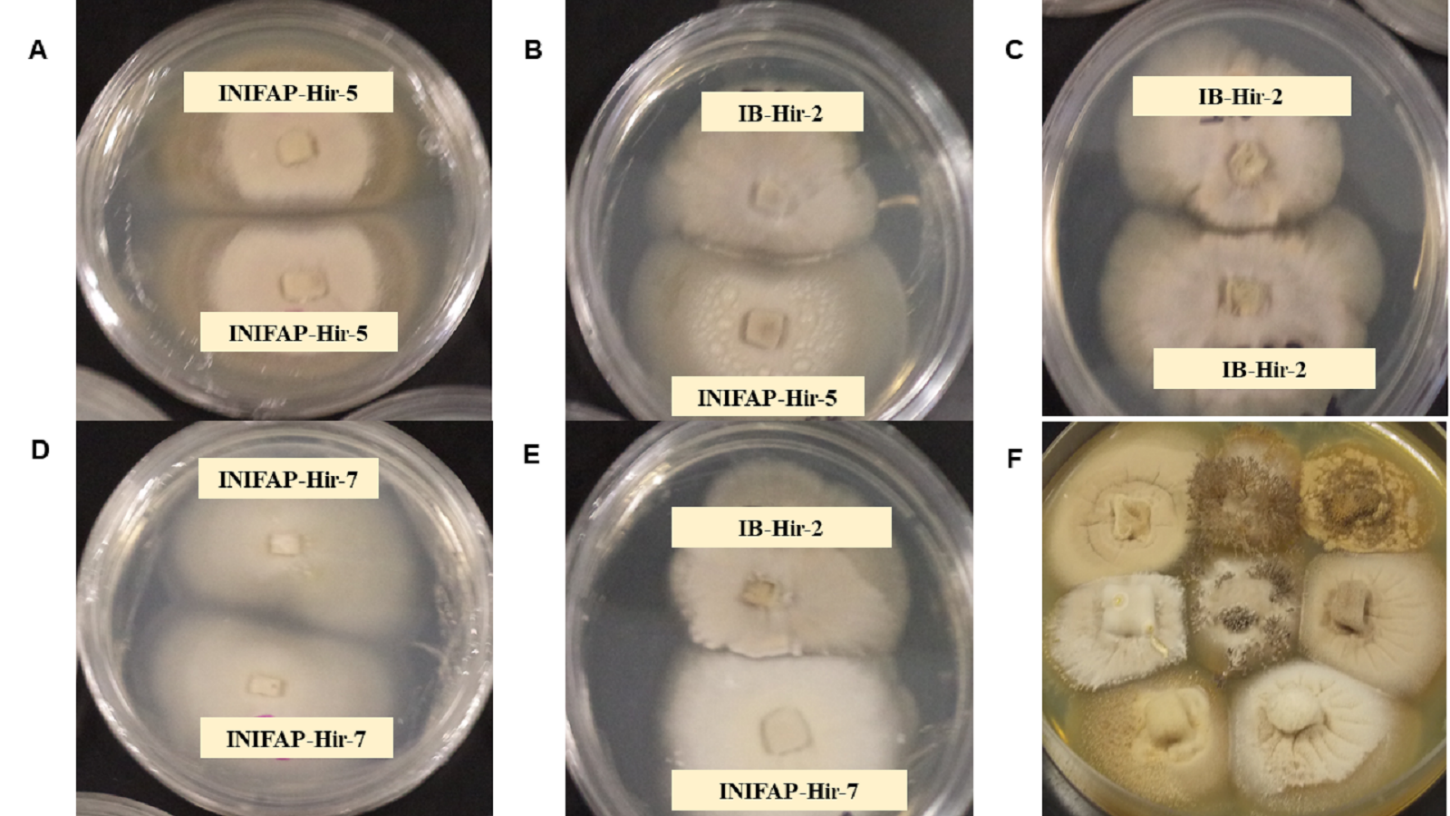
Mycelial compatibility among *Hirsutella citriformis* strains. (A, C & D) Mycelial compatibility between two colonies from one strain (INIFAP-Hir 5 vs INIFAP-Hir-5, IB-Hir-2 vs IB-Hir-2, or INIFAP-Hir 7 vs INIFAP-Hir-7, respectively). (B) Mycelial compatibility between the IB-Hir-2 vs INIFAP-Hir-5 strains. (E) Mycelial compatibility between the IB-Hir-2 vs INIFAP-Hir-7 strains. (F) Mycelial compatibility among the eight *H. citriformis* tested strains.

Thirty-six pairings were performed with the eight evaluated stains. All combinations resulted in a compatible reaction, where strains mycelia intermingled at the interaction zone. Both the compatibility between the same strain and the compatibility among strains, showed what appears to be a separation line in the colony border among them; nevertheless, this was only observed by the aerial mycelia since in the agar internal growth, mycelia combination among strains was observed. Cell death related to incompatibility or growth inhibitions due to antagonism were not detected among strains (Fig. 1).

### Anastomosis

**Figure 2:**
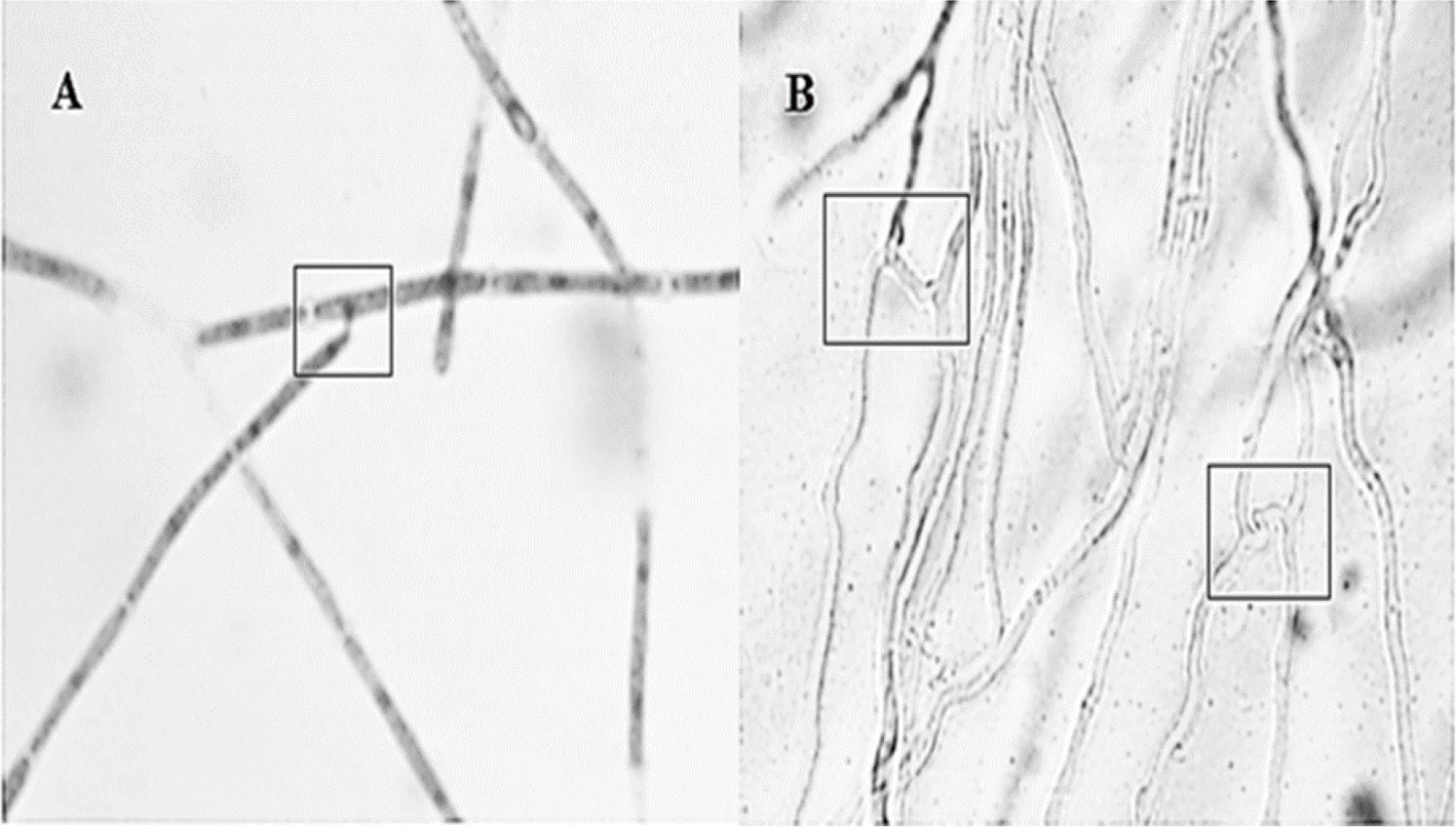
Different interactions observed by the *Hirsutella citriformis* strain INIFAP-Hir-1 anastomosis. (A) Anastomosis event occurred between hyphal tip with lateral portion of other hyphae; (B) anastomosis parallel bridges between hyphae.

Anastomosis experiments revealed all strain combinations performed anastomosis (Fig. 2). Anastomosis events occurred between hyphal tip with other hyphae lateral portion (first type) or by parallel bridges between hyphae cells (second type). The second type was the most frequent anastomosis event observed.

### Vegetative compatibility

**Figure 3:**
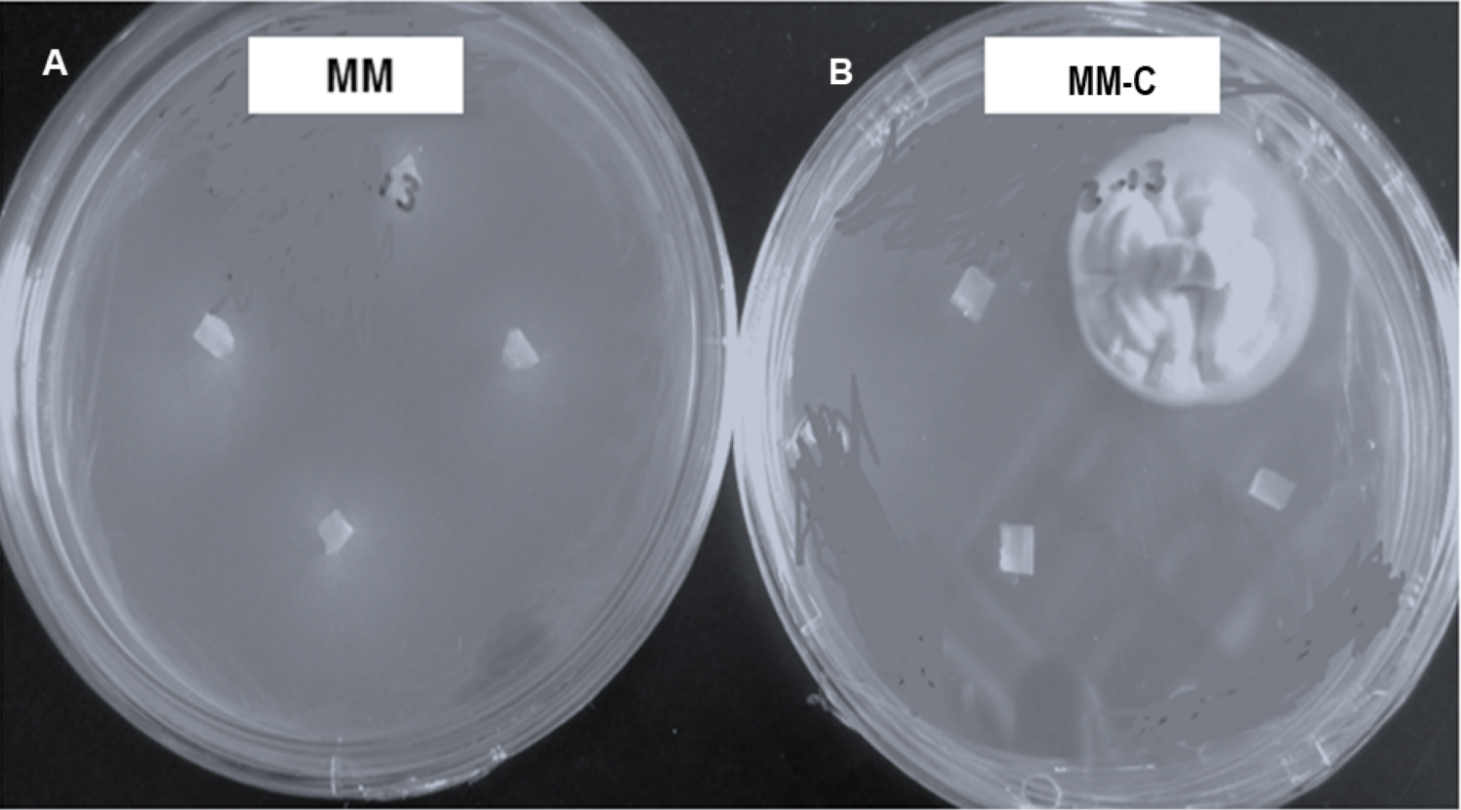
*Hirsutella citriformis* strain INIFAP-Hir-4 hyphal growth in restrictive media. (A) Wild-type strain growth in minimum medium (MM); (B) colony growth in chlorate minimum medium (MM-C).

Vegetative compatibility groups were observed, but all the colonies showing the strain typical growth of *nit* mutant were reported. All strains grew and produced non-dense mycelium in MM. In contrast, colonies developing on chlorate plates showed dense fluffy grown mycelium (Fig. 3).

Seventeen colonies grew on 2.5% chlorate MM-C plates, whereas only one colony (INIFAP-Hir2 strain) grew on 3% chlorate MM-C. INIFAP-Hir-1, INIFAP-Hir-5, INIFAP-Hir-6, and IB-Hir-1 strains did not grow at these chlorate concentrations. After mycelium samples of these strains were transferred from the MM-C to MM, all developed non-dense mycelia.

**Figure 4:**
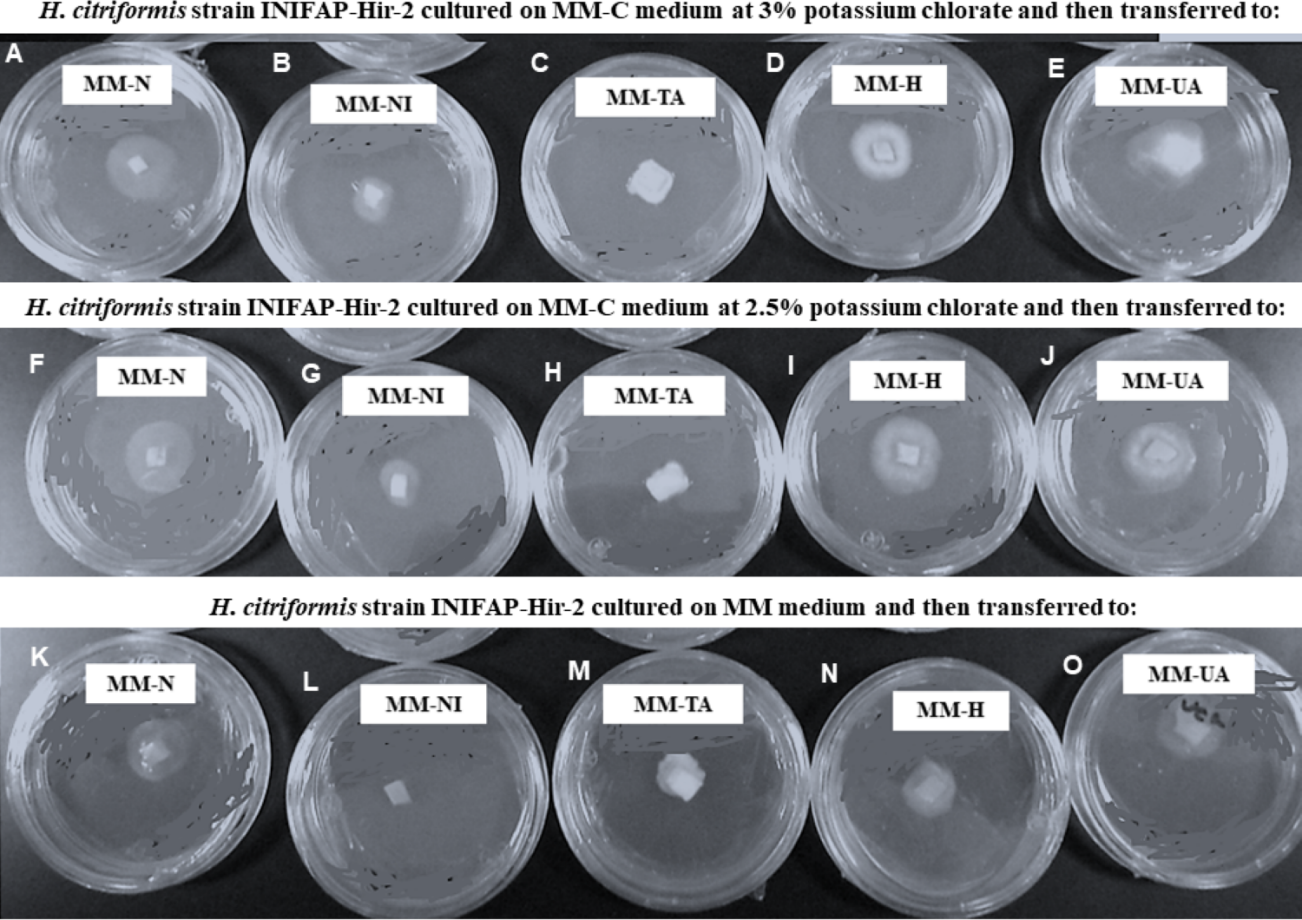
Phenotypic characterization of *Hirsutella citriformis* strain INIFAP-Hir-2 on selective media for *nit* mutants. Strain was exposed to chlorate minimum medium (MM-C) at 3% potassium chlorate, and transferred to minimal medium (MM) supplemented with the following nitrogen sources to characterize nit mutants, (A) 2 g/L nitrate = MM-N, (B) 0.5 g/L nitrite = MM-NI, (C) 1.6 g/L ammonium tartrate = MM-TA, (D) 0.2 g/L hypoxanthine = MM-H, and (E) 0.2 g uric acid = MM-UA. It was also exposed to MM-C at 2.5% potassium chlorate, and transferred to MM with the same nitrogen sources as described above, where (F) 2 g/L nitrate = MM-N, (G) 0.5 g/L nitrite = MM-NI, (H) 1.6 g/L ammonium tartrate = MM-TA, (I) 0.2 g/L hypoxanthine = MM-H, and (J) 0.2 g uric acid = MM-UA, or exposed to MM-C without potassium chlorate, and transferred to MM with the same nitrogen sources as described above, where (K) 2 g/L nitrate = MM-N, (L) 0.5 g/L nitrite = MM-NI, (M) 1.6 g/L ammonium tartrate = MM-TA, (N) 0.2 g/L hypoxanthine = MM-H, and (O) 0.2 g uric acid = MM-UA.

*H. citriformis* strain developed colonies were characterized upon different nitrogen sources utilization (Fig. 4). All strains produced dense mycelium on MM-TA, MM-H, and MM-UA, whereas non-dense mycelium was developed on MM-N and MM-NI. These wild-type strains produced similar macroscopic colonies than those isolated from MM-C when testing different nitrogen sources. It was then not possible to obtain *nit* mutants since the isolated colonies similarly grew as the wild strains on the different nitrogen sources media.

### Conidia and mycelium nuclear characterization

**Figure 5:**
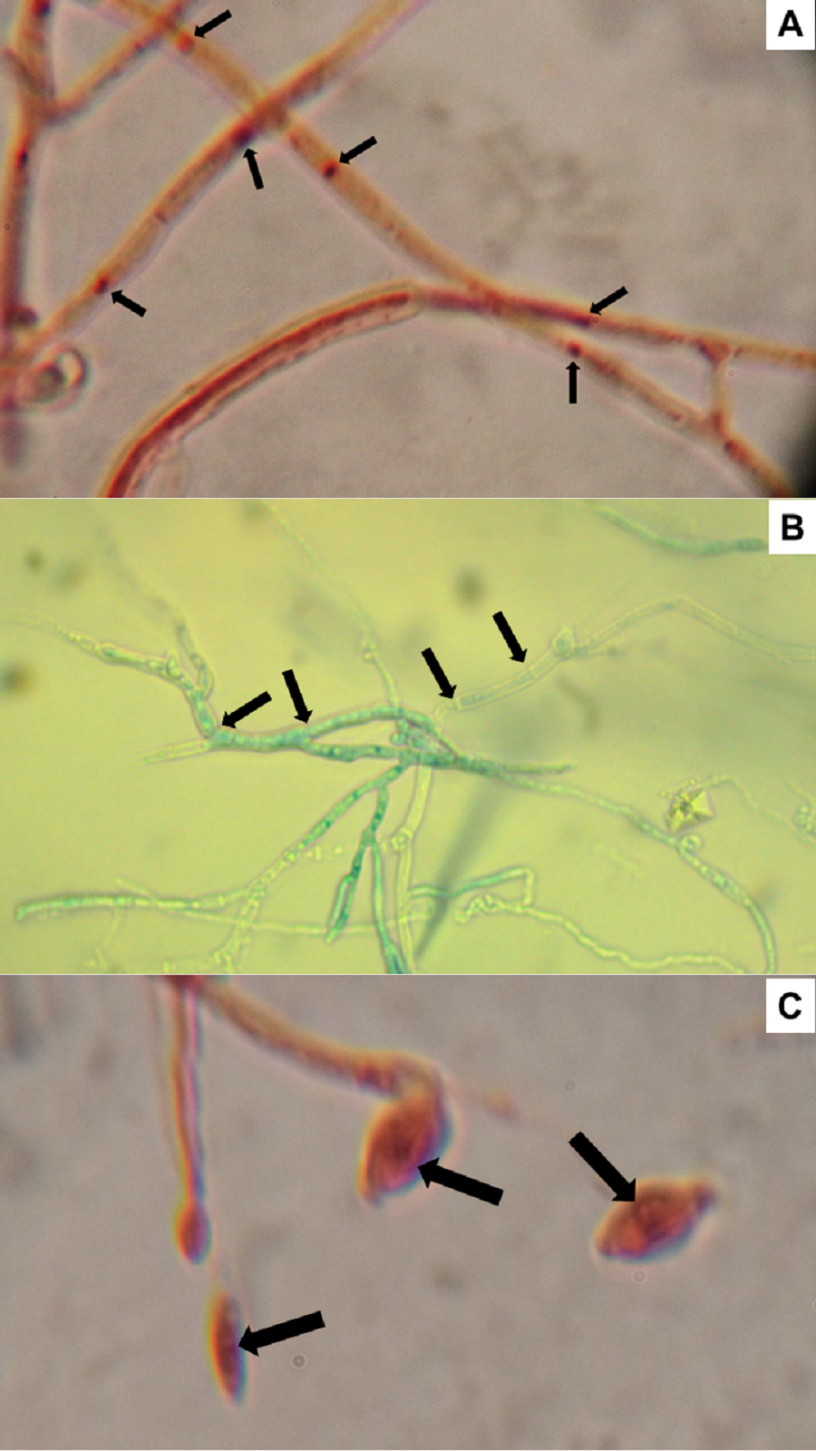
*Hirsutella citriformis* strain IB-Hir-2 hyphal growth. Strain was stained with (A) safranin O and potassium hydroxide, where arrows indicate nuclei; (B) lactophenol trypan blue (1.2 mL of liquified phenol, 10 mL of lactic acid, 8.8 mL of distilled water, and 0.02 g trypan blue), where arrows indicate mycelium septums; (C) IB-Hir-2 hyphal stained with safranin O and potassium hydroxide, where arrows indicate the nucleus inside a conidium.

Nuclei from 10 conidia and 10 cells of randomly selected *H. citriformis* strains were counted, from a location in the stain gradient, where nuclei and septa are distinctly observed. All strains showed a single nucleus in both conidia and mycelium (Fig. 5).

## Discussion

As previously described, *H. citriformis* has shown high mortality on *D. citri* adults exposed to conidia-bearing synnemata produced in vivo (adults *D. citri* cadavers) and cultured in vitro (Casique-Valdes et al., 2011; Meyer, Hoy & Boucias, 2007). *H. citriformis* persistence on adult *D. citri* from natural field infection tends to increase by humidity rate. Mycosed cadavers were persistent in the environment for more than two months (Hall et al., 2012). Therefore, *H. citriformis* has potential to be used for *D. citri* (citrus psyllid) and *B. cockerelli* biocontrol, insect vectors of diseases that cause significant agriculture problems (Subandiyah et al., 2000; Meyer, Hoy & Boucias, 2007; Casique-Valdes et al., 2011; Pérez-González et al., 2016a; Pérez-González, Sandoval-Coronado & Maldonado-Blanco, 2016b). Nevertheless, it is a fungus difficult to isolate and culture, for which has been scarcely studied.

Mycelial compatibility results were similar than those reported by Remesal et al. (2012), who showed that *Sclerotium rolfsii* (Sacc.) West. strains were self-compatible. Similarly, Akram et al. (2008) observed 58% mycelial compatibility among *Sclerotinia sclerotiorum* (Lib.) isolates. All strains developed anastomosis with themselves and those considered self-compatible. Anastomosis with nuclei fusion and exchange of genetic information is a common event reported for plant pathogenic fungi such as *Colletotrichum lindemuthianum* (Sacc. Et. Magn.) Scrib. (Rodríguez-Guerra, Ramírez-Rueda & Simpson, 2004), *Rhizoctonia solani* Kühn (Chávez-Barragán et al., 2011), *Monilinia fruticula* (De Cal, Egüen & Melgarejo, 2014), and among several entomopathogenic fungi such as *Metarhizium anisopliae* (Metschnikoff) Sorokin (Tinline & Noviello, 1971; Messias & Azevedo, 1980; Paccola-Meirelles & Azevedo, 1991; Sirisha, Gurvinder Kaur & Padmini Palem, 2010), and *Beauveria bassiana* (Bals.-Criv.) Vuill. (Yurchenko, Zakharov & Levitin, 1974; Paccola-Meirelles & Azevedo, 1991; Sirisha, Gurvinder Kaur & Padmini Palem, 2010).

Strains grown in MM produced non-dense mycelium, unlike fungi such as *Colletotrichum kahawe* J.M. Waller & Bridge (Varzea, Rodrigues & Lewis, 2002) and *Verticillium dahlia* Kleb. (Dervis et al., 2009), which developed dense mycelium on MM.

In order to phenotypically characterize strains upon *nit* mutant types, MM was supplemented with ammonium tartrate (MM-TA), nitrate (MM-N), nitrite (MM-NI), hypo-xanthine (MM-H) or uric acid. Type *nit1* mutant does not use nitrate or hypoxanthine; mutant type *nit3* does not use nitrate, nitrite, hypoxanthine or uric acid; mutant type *nit* M does not use nitrate or hypoxanthine; mutant type *nnu* does not use nitrate, nitrite or hypoxanthine; whereas mutant type *nit* 4 does not use nitrate (Cove, 1976; Garrett & Amy, 1978).

Colonies developed from *H. citriformis* strains on MM-C transferred to MM with nitrate as the sole source of nitrogen, showed thin, expansive, and appressed growth. This type of growth is similar than that reported by *nit* mutants. Nevertheless, wild strains showed similar growth on MM with nitrate.

Furthermore, mycelium developed by the strains cultured on MM-C (2.5% and 3% chlorate) showed that *Hirsutella* strains were stimulated to develop dense mycelium colonies. All colonies from MM-C as well as the wild strains, presented similar growth on different nitrogen sources (dense mycelium on organic nitrogen sources and non-dense mycelia on inorganic nitrogen sources). Results provided evidence of the expected compatibility among tested isolates, thus indicating that selected strains are closely related.

Enzymes production studies developed on this fungus did not show significant correlation with its pathogenicity (Cortez-Madrigal et al., 2014; Quesada-Sojo & Rivera-Méndez, 2016; Pérez-González & Valdes-Gonzalez, 2017). However, it has not been determined whether the pathogenic metabolism relies on transposon or mycovirus elements that may play a role as observed by *B. bassiana* (Kotta-Loizou & Coutts, 2017; Pitaluga et al., 2020). Results from our study may help to better understand this fungus metabolism. In this regard, compatibility results might be a factor to determine whether each strain characteristics are related to growth and development on culture media, as well as pathogenicity in the laboratory and in the field.

Future research on *H. citriformis* is underway to better understand if nuclei exchange between strains or extrachromosomal material exchange affects its pathogenicity. In addition, dissemination, population richness, and infection mechanism field studies on this fungus are required, since limited research related to the presence of the fungus parasitizing the insect has been performed.

## Conclusions

*H. citriformis* strains tested in this study presented compatible mycelium and anastomosis among them. Evaluated strains showed one nucleus per conidium and mycelium section. All tested strains developed dense mycelium in inorganic nitrogen sources. Results also indicated that genetic information may be exchanged by asexual reproduction (hyphal anastomosis) between different *H. citriformis* strains isolated from *D. citri*. It was not possible to obtain *H. citriformis nit* mutants at the chlorate concentrations tested. To date, this is the first report describing the mycelial compatibility, anastomosis occurrence, and number of nuclei present in hyphae and conidia among *H. citriformis* strains.

## Abbreviations list

Fe(NH_4_)_2_(SO_4_): Ammonium iron(II) sulfate
MM: basal medium
cm: centimeter
CuSO_4_: copper sulfate
d: day
g: gram
°C: degrees Celsius
MnSO_4_: manganese sulfate
mL: milliliter
mm: millimeter
MM-C: MM chlorate
MM-TA: MM ammonium tartrate
MM-N: MM nitrate
MM-NI: MM nitrite
MM-H: MM hypoxanthine
MM-UA: MM uric acid
H_3_PO_4_: phosphoric acid
KClO_3_: potassium chlorate
KOH: potassium oxide
PDA: potato dextrose agar
NaMoO_4_: sodium molybdate
H_2_O: water
WA: water agar
WAD: water agar dextrose
wk: week
ZnSO_4_: Zinc sulfate

## Additional Information and Declarations

### Data Availability

The following information was supplied regarding data availability: Data are available in Figs. 1–5 and any available numeric data are reported in the Results section.
